# A Scoping Review of Sexual Violence Events Perpetrated Against Older People

**DOI:** 10.1177/15248380241265387

**Published:** 2024-07-31

**Authors:** Madeline Lee, Nadine McKillop, Emily Moir

**Affiliations:** 1 University of the Sunshine Coast, Sippy Downs, QLD, Australia

**Keywords:** sexual violence and abuse, older people, criminal events, environmental criminology, scoping review

## Abstract

Although 9 million older adults are estimated to be victims of sexual violence and abuse (SVA) globally each year, this population is largely overlooked in criminological research. Given the known impacts of SVA, particularly for older victims, enhanced understanding of incident characteristics, and how these events unfold, is crucial to improving prevention and response. Guided by environmental criminology perspectives, a scoping review was conducted to assess the extent to which SVA events against older people have been empirically examined to gain an understanding of the immediate circumstances in which incidents occur and how this can inform early intervention and prevention. Eight databases were searched, with records included if they reported characteristics of SVA events occurring in older adulthood, were original, peer-reviewed, empirical research, and published in English. After screening 1,278 records, 33 studies were included for review. Findings demonstrate that considerable attention has been given to understanding who is involved in SVA events and what typically occurs, however, there is a dearth of information regarding when and how these crimes are perpetrated, and the situational factors influencing offending. Resultingly, prevention recommendations largely focus on actors (e.g., victims, perpetrators, witnesses), rather than the environment in which abuse occurs, emphasizing significant gaps in understanding and response to this growing issue.

According to the World Health Organization (WHO, 2022), by 2050, the proportion of people aged over 60 years is expected to double, while those aged over 80 years will likely triple, reaching approximately 426 million. Though aging is a diverse and non-linear process (WHO, 2022), individuals often encounter cognitive and physiological changes throughout older adulthood which can increase their vulnerability toward violence and abuse (Chopin & Beauregard, 2020a; Ramsey-Klawsnik, 2003). For instance, United Nations (n.d.) data suggests more than 46% of older adults have a disability, while an estimated one-third of individuals over 85 years of age live with a form of dementia (National Institute on Aging, 2022). Literature shows that these characteristics are primary risk factors for sexual violence and abuse (SVA) among older adults (Bows, 2018; Dong, 2015; Ramsey-Klawsnik et al., 2008). With upward trends in global population aging, it is expected that SVA rates among older adults will increase comparably, emphasizing the need for more empirical understanding of this issue to enact informed and effective responses and reduce the extent and impacts of this problem.

## Prevalence

Yon et al. (2017) estimate that global SVA prevalence among community-dwelling older adults is 0.9%, equating to approximately 9 million older victims each year.^1^ Accounting for projected population increases, this figure could rise to almost 19 million older victims annually if appropriate action is not taken to combat rising prevalence. Moreover, the convergence of vulnerabilities within aged care institutions sees SVA rates in these settings being more than double that of the community (1.9%; Yon et al., 2018), further asserting the criticality of understanding and addressing this complex issue.

Concerningly, these figures likely underestimate the true number of SVA incidents due to significant underreporting. The older population, in particular, faces considerable barriers to disclosure and reporting, such as understanding that certain acts constitute abuse, recall difficulties, communication challenges, dependency on an abuser, and feelings of shame, self-blame, and fear (Fileborn, 2017; Goldblatt et al., 2022; Ramsey-Klawsnik & Teaster, 2012). Moreover, ageist stereotypes suggest older people cannot be victims of SVA as they are neither sexually active nor attractive, having a detrimental impact on recognition and response to suspected incidents, or disclosures, at both an individual and societal level (Goldblatt et al., 2022). Consequently, prevalence, data accuracy, and subsequent understanding of the issue may be underacknowledged.

## Past Reviews

Over the past decade, numerous reviews have been conducted on the broader topic of elder abuse, however, few have systematically evaluated literature on SVA perpetrated against older people (e.g., Bows, 2018; Fileborn, 2017; Hand et al., 2022; Malmedal et al., 2015; Nobels et al., 2020; Smith et al., 2018). Of these, most focus on understanding individual characteristics and risk factors for incidents, along with barriers to reporting, and prevention recommendations. While these concepts are important for informing response, it is argued that more diverse opportunities for proactive prevention can be gained through comprehensive understanding of the SVA event itself. In their recent narrative review on the sexual victimization of older adults, Georgoulis et al. (2024) emphasized the importance of understanding this issue from a criminological perspective (i.e., routine activity approach), summarizing victim vulnerability factors, offender motivations, and assault characteristics. Yet, deeper consideration of how these incidents manifest and unfold in time and place is needed to enhance prevention efforts, integrating knowledge of individual-level risk and environmental factors that facilitate or induce offending behavior (Wortley & Townsley, 2017). Consideration of this issue from an event perspective presents the opportunity to examine literature through an environmental criminology lens, thus enriching and expanding current understandings.

## Environmental Criminology

Environmental criminology purports that the immediate environment is integral to offending, whereby situational factors present opportunities for criminal behavior, or may even induce offending (Sidebottom & Wortley, 2016; Wortley, 1997; Wortley & Townsley, 2017). Three theoretical approaches underpin environmental criminology; the routine activity approach, rational choice perspective, and crime pattern theory. Collectively, these theories contribute to a more comprehensive understanding of the criminal event, and the role situations play in enabling crime and victimization, generating opportunities for proactive prevention (Beauregard & Chopin, 2022; Sidebottom & Wortley, 2016; Wortley & Townsley, 2017), which is beneficial for exploring SVA events perpetrated against older people.

### Routine Activity Approach

The routine activity approach purports that when motivated offenders and suitable targets converge in time and place, in the absence of capable guardians, crime has a higher likelihood of eventuating (Cohen & Felson, 1979). Centered on victimization, this perspective has been previously applied to explain abuse against older people (e.g., Beauregard & Chopin, 2022; Blundell et al., 2022; Chopin & Beauregard, 2020a; Payne & Gainey, 2006), benefiting extant knowledge through recognition of the lifestyle characteristics and inherent vulnerabilities that may increase risk within this population. For example, aging often sees decreases in physical strength and agility, thereby reducing capacity for self-protection and resistance against victimization (Ramsey-Klawsnik, 2003), while cognitive decline may influence cognizance of abuse and subsequent reporting behaviors (Vierthaler, 2008). Taken together, these characteristics may increase perceived victim suitability, whereby perpetrators may purposefully seek out people who can be easily overpowered and are less likely to report (Vierthaler, 2008).

More recently, Eck (2003) extended the routine activity approach to include “controllers” whose role is to prevent and deter potential crime. Bounding the three original crime elements, controllers include guardians, who mitigate target risk; handlers, who regulate offender behavior; and, managers, who govern events within the places under their control (Clarke & Eck, 2016; Eck, 2003). Certainly, guardianship has been empirically linked with sexual offense deterrence and cessation (Cook et al., 2021; Leclerc et al., 2015; McKillop et al., 2021), including within institutional settings (Lockitch et al., 2022). Yet, older people routinely experience lack of capable guardianship, such as through social isolation and living alone (Bows, 2018; Lazar, 2019; Murphy & Winder, 2016), or in institutional settings where staff numbers are often deemed inadequate and supervision is lacking (Ramsey-Klawsnik & Teaster, 2012; Ramsey-Klawsnik et al., 2008). Ultimately, these issues can be seen to further manifest opportunities for crime to be pursued against potentially vulnerable older adults.

### Rational Choice Perspective

Complimentary to the routine activity approach, the rational choice perspective focuses on “motivated offenders”, suggesting that potential perpetrators successfully achieve criminal outcomes by making a series of purposive decisions throughout offense commission, influenced by situational factors and perceptions of benefit (Cornish & Clarke, 2017). Although application of this perspective to explain sexual offending has received criticism due to perceptions of irrationality and impulsivity throughout offense commission (Cornish & Clarke, 2017), research conducted by Beauregard and Leclerc (2007) refutes this argument, finding these offenders frequently engage in cost-benefit analysis, with decisions dynamically influenced by situational factors.

This concept is further validated through the work of Wortley (1997, 1998), who purported that the immediate environment can play an active role in encouraging or inducing criminal behavior. As such, situational cues may prompt certain behaviors, exert pressure to conform to social expectations, permit criminality through weakened self-control or moral restraints, or provoke responses through emotional arousal. The rational choice perspective is therefore critical to examining SVA events against older people to identify situational cues evoking decision-making and inducing criminal behavior, which may help direct prevention and intervention efforts.

### Crime Pattern Theory

While routine activity and rational choice perspectives focus on victim vulnerability and offender decision-making, crime pattern theory emphasizes *where* and *when* incidents are most likely to occur, drawing attention to the convergence of crime elements in time and place (Brantingham et al., 2017; Eck & Weisburd, 1995; Sidebottom & Wortley, 2016). The theory suggests that crime is not random, but rather follows spatial and temporal patterns, as determined by the routine activities of potential offenders (Brantingham et al., 2017; Sidebottom & Wortley, 2016). As such, perpetrators are more likely to offend in areas they frequent, seeing crime clustered around key activity spaces (Brantingham et al., 2017; Eck & Weisburd, 1995; Leclerc, Chiu, Cale, & Cook, 2016). Identification of where and when specific crimes are most likely to occur can then allow for more targeted prevention measures in high-risk times and places.

### Situational Crime Prevention

A key advantage of environmental criminology is that it considers all aspects of crime (victim, perpetrator, and place) to provide insights into where, when, and how specific crimes are perpetrated, therefore informing proactive prevention initiatives (Leclerc, Chiu, & Cale, 2016). Consequently, prevention efforts in this space are often concentrated on altering criminogenic environments to reduce opportunity; a method known as situational crime prevention (SCP; Clarke, 2017; Clarke & Eck, 2016; Felson & Eckert, 2018). The SCP framework is recognized as a comprehensive and diversely applicable approach, offering a catalog of techniques to increase effort and risk, reduce rewards and provocations, and remove excuses within potentially volatile environments (Clarke, 2017; Clarke & Eck, 2016; Felson & Eckert, 2018). SCP’s inextricable links with environmental criminology signify the need for a comprehensive understanding of the criminal event—the actors, decisions, behaviors, and environment—to ensure prevention efforts are appropriately informed and therefore likely to be effective (Clarke, 2017; Wikstrom, 2007).

Due to their proactive approach to preventing crime, strategies underpinned by SCP are often classified as primary prevention according to the public health model—a systematic framework informing holistic prevention and intervention of SVA at primary, secondary, and tertiary levels (Quadara et al., 2015). Pertinent to environmental criminology and the criminal event, primary and secondary prevention measures are recognized for their role in actively deterring or intervening early in crime events (Atkinson & Roberto, 2023; Brantingham & Faust, 1976; Quadara et al., 2015). Primary initiatives are often implemented at a community level, targeting origins of abuse and preventing harm before it occurs, such as through education, environmental design, and SCP, while secondary strategies aim to quickly identify at-risk individuals or settings and intervene before harm escalates (Atkinson & Roberto, 2023; Brantingham & Faust, 1976; Quadara et al., 2015). While focus at this level is generally targeted toward potential perpetrators, Atkinson and Roberto (2023) suggest secondary prevention also encompasses education and training for potential guardians to recognize and respond to signs of abuse, therefore facilitating early intervention to forestall harm and mistreatment. Enhanced understanding of the criminal event, as guided by environmental criminology, is therefore crucial for directing targeted and holistic prevention efforts.

## The Current Study

To date, no known review has yet assessed the extent to which older-victim SVA event characteristics have been empirically examined within extant literature. The current study therefore explored factors associated with these events to gain a more comprehensive understanding of the immediate circumstances in which incidents occur, and how this can inform early intervention and prevention. To collate data and enhance interpretations, environmental criminology perspectives (i.e., routine activity, rational choice, and crime pattern theories) guided the review to determine who is involved in SVA events, what typically occurs, where and when offenses usually take place, and how they are perpetrated. This approach has been previously employed to review extant literature on SVA events involving child and younger adult victims (Leclerc, Chiu, & Cale, 2016; Leclerc, Chiu, Cale, & Cook, 2016), however, its application to older adults remains underexplored, presenting an opportunity to contribute to scholarship in this field and provide deeper understanding of the factors influencing sexual offenses. With specific interest in how SVA events involving older victims can be prevented, this review also documented prevention recommendations made within extant literature, according to the public health model, to determine current approaches and inform future directions.

In sum, this scoping review examined the breadth of literature on SVA perpetrated against older people, summarized reported event characteristics and proactive prevention recommendations, and identified gaps in knowledge and understanding.

## Methodology

This study followed the five stages outlined within Arksey and O’Malley’s (2005) methodological framework for scoping reviews. Refinements recommended by Levac et al. (2010) and Daudt et al. (2013) were also incorporated, where appropriate (e.g., ensuring a clearly articulated scope of inquiry and that all team members contribute to study identification and selection stages, following an iterative process).

### Identifying the Research Question

As Levac et al. (2010) recommend, specifying the scope of inquiry, target population, and outcomes of interest are crucial when developing research questions. Guided by environmental criminology, the purpose of this review was to summarize reported characteristics of SVA criminal events involving older victims and determine recommended avenues of prevention, relative to immediate circumstances of the offense (e.g., primary and secondary prevention strategies). Therefore, the following research question was formulated:

What does literature report about the nature and dimensions of SVA events involving older victims, and what prevention recommendations are made?

### Identifying Relevant Studies

Relevant studies were identified via three avenues. First, the search string “((“sexual violence” OR “sexual assault” OR “sexual abuse” OR rape) AND (old* OR elder* people) NOT (youth OR child* OR adolescen* OR student OR “young* adult*”))” was entered across eight databases, with adjustments to database specifications made when necessary. Databases included Informit, ProQuest Criminal Justice Database, Scopus, PubMed, Web of Science, EBSCO Criminal Justice Abstracts, PsychNet (APA), and Google Scholar. Results were filtered to display entries published from the year 2000 onwards (up to and including the search date—November 15, 2023) to capture a broad range of topical literature within this field. All results^2^ were exported to referencing management software, Endnote, before being imported into Covidence for screening. Additionally, reference lists of included studies were reviewed, with additional sources added to the selection, along with articles not captured during database searches but known by the research team to be applicable.

### Study Selection

**Figure 1.**
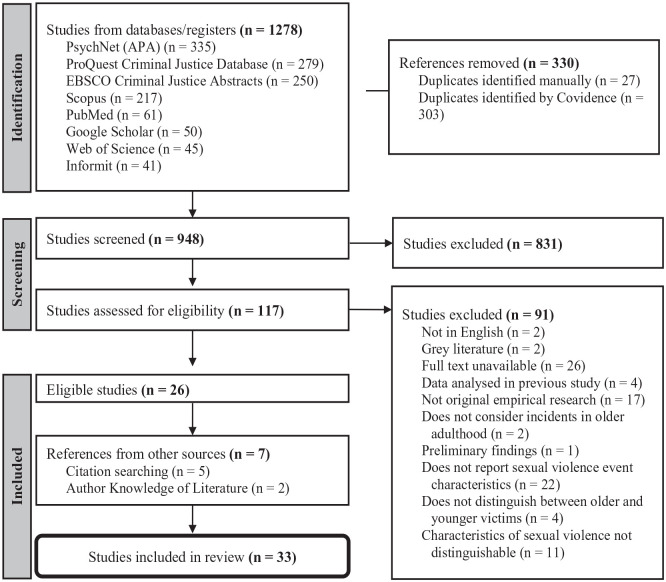
Scoping review screening process.

As shown in Figure 1, database searches returned 1,278 results. Once imported to Covidence, 330 duplicate studies were removed, leaving 948 studies for screening. First, titles and abstracts were screened to determine suitability, with studies included at this stage if they initially appeared to examine incidents of SVA perpetrated against older people and were peer reviewed. With varying definitions of “old” age across jurisdictions, cultures, and research methodologies (Lea et al., 2011; Moir et al., 2017), no age parameters were imposed for this review to ensure the scope included a broad range of applicable literature. Any clearly identifiable review articles (e.g., scoping, systematic, and book) were excluded at this stage to avoid potential double-reporting of original data. Ultimately, 831 studies were deemed irrelevant, leaving 117 for full-text review.

All 117 documents were blind reviewed by 2 reviewers, with decisions based on a comprehensive list of exclusion criteria (see Table 1). Conflicts were discussed among the research team, and resolved by a third reviewer, to determine final suitability. Five additional studies were located through reference list searching, and 2 were added based on author knowledge of literature, bringing the final number of included studies to 33.

**Table 1.** Full-Text Screening (Exclusion Criteria).

• Does not report SVA event characteristics • Characteristics of SVA events not distinguishable (when reported among other abuse types) • Does not consider incidents in older adulthood • Does not distinguish between older and younger victims • Not original empirical research • Preliminary findings (full results in later article, also included in screening) • Data analyzed in previous study • Gray literature • Not in English • Full text unavailable

*Note*. SVA = sexual violence and abuse.

### Charting the Data

Two reviewers conducted extraction on all 33 included studies, with a third ensuring consensus was reached. Data was extracted according to key principles of environmental criminology to gain an understanding of the circumstances of identified criminal events (i.e., who, what, when, where, and how), as similarly evidenced in previous SVA research (see Leclerc, Chiu, & Cale, 2016; Leclerc, Chiu, Cale, & Cook, 2016). To assess how these characteristics informed prevention, recommendations were categorized according to the public health model, focusing on primary and secondary strategies pertinent to the immediate event. Additionally, information on study methodology, sample characteristics, country of research, theoretical framework, and definition of “older person” were extracted.

## Results

### Overview of Studies

Table 2 provides an overview of included studies. Of the 33 studies reviewed, majority were conducted in the United States (*n* = 15) and the United Kingdom (*n* = 7). Most studies implemented a quantitative research design (*n* = 25), with five using mixed methods and three analyzing data qualitatively. When considering data samples, a large proportion used reported SVA cases involving an older victim (*n* = 22), while five surveyed older people directly about their experiences of abuse, often to gauge broad prevalence of multiple abuse types. Where study setting was reported, majority explored mixed settings (i.e., both community and institutional locations; *n* = 16), though seven studies exclusively focused on incidents within the community, and six in institutions only.

**Table 2.** Overview of Included Studies.

Citation	Design and Theory	Sample and Country	Setting	“Older” (Years)
Almond et al. (2022)	Quantitative	143 SVA cases reported to police between 2003 and 2017 in Canada	Mixed	60+
Alon et al. (2018)	Quantitative	161 Surveys completed by social workers and nurses in Israel	Mixed	
Baker et al. (2009)	Quantitative; Rose and Killien’s (1983) risk and vulnerability model	198 Women who presented for a sexual assault exam in the United States	Mixed	50+
Ball and Fowler (2008)	Quantitative	1,062 SVA offenses (*n* *=* 16 older victims) recorded between April 1999 and June 2004 in the United Kingdom	Community	60+
Bows and Westmarland (2017)	Quantitative	87,230 SVA cases (*n* = 655 older victims) reported to 45 police forces between 2009 and 2013 in the United Kingdom	Mixed	60+
Budd and Liddic (2021)	Quantitative	471,305 SVA cases (*n* = 12,620 older victims) reported to law enforcement from 1992 to 2015 in the United States	Mixed	60+
Burgess and Phillips (2006) ^a^	Quantitative	284 SVA cases investigated by law enforcement in the United States	Mixed	60+
Burgess et al. (2008) ^a^	Quantitative	284 SVA cases reported to APS or CJSin the United States	Mixed	60+
Cannell et al. (2014)	Quantitative	108,765 People (*n* = 24,343 older adults; *n* = 222 older SVA victims) from the 2005 Behavioral Risk Factor Surveillance System survey in the United States	Community	60+
Chopin and Beauregard (2020a)	Quantitative; rational choice and routine activity approaches	1,829 Extrafamilial SVA cases (*n* = 130 older) reported to police between 1979 and 2014 in French Territory	—	65+
Chopin and Beauregard (2020b)	Quantitative; routine activity approach	569 Extrafamilial sexual homicide cases reported to authorities in Canada and France	—	65+
Del Bove et al. (2005)	Quantitative	212 Clients presenting to a hospital-based Sexual Assault Care Center between 1992 and 2002 in Canada	Mixed	55+
Eckert and Sugar (2008)	Quantitative	2,399 Women (*n* *=* 102 older) reporting to an urban emergency department for SVA evaluation in the United States	Mixed	55+
Filipska et al. (2019)	Quantitative	Survey of 200 hospital in-patients (*n* = 17 older SVA victims) in Poland	—	60+
Fisher and Regan (2006)	Quantitative	Telephone survey with 842 older women (*n* = 28 SVA victims) in the United States	Community	60+
Jeary (2004)	Qualitative	52 SVA cases, semi-structured interviews with correctional case managers, and focus groups with managers of care facilities in the United Kingdom	Mixed	60+
Jeary (2005)	Qualitative	52 SVA cases (*n* *=* 54 victims), interviews with practitioners and 6 prisoners, focus groups with managers of residential care homes/domiciliary care provision in the United Kingdom	Mixed	60+
Jordanova Peshevska et al. (2014)	Quantitative	Survey of 960 older adults (*n* = 13 SVA victims) in the Republic of Macedonia	Community	65+
Lazar (2019)	Quantitative, case law analysis	109 SVA court cases (*n* = 147 older women) between 2000 and 2018 in the United States	Mixed	60+
Lea et al. (2011)	Quantitative	106 SVA cases (*n* *=* 53 older) reported in the Serious Crime Analysis Section system in the United Kingdom	—	60+
Lee et al. (2019)	Quantitative	39 Older adults who attended a Sexual Assault Referral Center for a Forensic Medical Examination, compared with 736 clients aged 18–69 years, in the United Kingdom	Mixed	70+
Murphy and Winder (2016)	Qualitative	Interviews with five men in custody for an SVA offense against an older victim in the United Kingdom	Community	55+
Nobels et al. (2021)	Quantitative	Interviews with 100 older adults (*n* = 7 SVA victims) receiving in-patient treatment at three old age psychiatry wards in Belgium	Mixed	70+
Nóbrega Pinto et al. (2014)	Quantitative	Forensic reports and judicial outcomes of 14 suspected SVA cases in Portugal	Mixed	65+
Payne and Gainey (2006)	Mixed methods; routine activity approach	801 Abuse cases (*n* = 78 SVA) reported in the Medicaid Fraud Report between January 1997 and May 2002 in the United States	Institutional	—
Payne (2010)	Mixed methods; routine activity and life-course theories	127 SVA cases, compared with 314 physical abuse cases selected from Medicaid Fraud Reports in the United States	Institutional	—
Qu et al. (2023)	Quantitative	Telephone survey of 7,000 older people (*n* = 70 SVA victims) in Australia	Community	65+
Ramsey-Klawsnik et al. (2008)	Mixed methods	124 Alleged SVA cases (33 confirmed) reported to APS between May and October 2005, and follow-up interviews with 15% of personnel who investigated the cases in the United States	Institutional	60+
Safarik et al. (2002)	Mixed methods	128 Sexual homicide cases (110 responsible offenders), sourced from the National Center for the Analysis of Violent Crime in the United States	Community	60+
Smith et al. (2019)	Quantitative	28 Alleged SVA incidents reported to the Clinical Forensic Medicine service between 2000 and 2015 in Australia	Institutional	65+
Teaster and Roberto (2004)	Quantitative	82 SVA reports investigated by APS between July 1996 and June 2001 in the United States	Mixed	60+
Teaster et al. (2007)	Quantitative	26 SVA cases involving an older male, investigated by APS between May 2005 and October 2005 in the United States	Institutional	50+
Teaster et al. (2015)	Quantitative; Democratic governance theory	64 SVA cases investigated by APS and regulatory agencies over a 6-month period in 2005 in the United States	Institutional	65+

*Note*. SVA = sexual violence and abuse; APS = Adult Protective Services; CJS = Criminal Justice System

a Studies used similar samples, though analyzed different dimensions of SVA, hence both are included.

#### Theoretical Framework

Use of theory to inform empirical research was noticeably absent within included studies. Only six integrated an identified theoretical framework (Baker et al., 2009; Chopin & Beauregard, 2020a, 2020b; Payne, 2010; Payne & Gainey, 2006; Teaster et al., 2015), with most (*n* = 4) falling under environmental criminology (e.g., routine activity approach, and rational choice perspective).

#### Definition of “Older Person.”

Significant variance was noted in the definition of an “older person,” with age classifications ranging from 50+ years (*n* = 2) to 70+ years (*n* = 2). Nonetheless, most studies used a minimum age of 60 (*n* = 16) or 65 (*n* = 7) years.

### Who Is Typically Involved in Incidents of SVA Against Older People?

#### Victims

An overwhelming proportion of studies reported older SVA victims are mostly female (*n* *=* 28), with more than 70% identifying as White (*n* *=* 14; see Supplemental Appendix A). A single study explored sexual assaults against older male victims exclusively (Teaster et al., 2007), though Ramsey-Klawsnik et al. (2008) and Payne (2010) similarly found higher rates of male victimization within institutional settings (23% and 33%, respectively). Where average age was recorded (*n* = 14), most studies (*n* = 9) stated victims were typically aged in their 70s, rising slightly in institutions (79–83 years average). The oldest recorded victim age was 101 years (Almond et al., 2022; Ramsey-Klawsnik et al., 2008; Teaster et al., 2015), though 18 studies included victims aged 90 years or above. Being single or widowed was common, and of the studies reporting living arrangements, most indicated that community-dwelling victims often lived alone.

Over half (*n* = 17) of all reviewed studies reported victims experienced neurological or physical limitations. Cognitive impairment, primarily dementia, was prevalent among victim samples (*n* = 13 studies), with rates varying between 13% (Lazar, 2019) and 73.9% (Smith et al., 2019). Predictably, all institution-based studies involved cognitively impaired victims, where rates were comparatively higher (36% minimum; Payne & Gainey, 2006). Physical limitations, such as disability, frailty, and mobility issues were also frequently identified (*n* = 13), with many studies reporting between 45% and 77% of victims were either non-ambulatory or required assistance with activities of daily living (Ramsey-Klawsnik et al., 2008; Smith et al., 2019; Teaster & Roberto, 2004; Teaster et al., 2007, 2015).

#### Perpetrators

Where gender was recorded (*n* = 18), most studies reported all male perpetrators, with the exception of Teaster et al. (2007) who found that 42% of alleged perpetrators were female (though only one female-perpetrated case was ultimately substantiated). In all studies reporting perpetrator ethnicity (*n* = 10), majority were White. Contrary to victims, perpetrator age varied significantly, with Budd and Liddic (2021) identifying the broadest range (10–99 years). On average, perpetrators were aged between 30 and 50 years, though older offenders were still regularly reported.

Six studies provided data on perpetrator impairments. In particular, mental impairment or illness was prominently reported (*n* = 5 studies), found to be as high as 70.5% in cases investigated by Adult Protective Services (Burgess et al., 2008). Three studies described physical impairment, with a rate of 28.1% identified in community settings (Qu et al., 2023) and up to 65.7% in institutional settings (Burgess et al., 2008). Moreover, 9 studies specified perpetrators were substance users and 10 stated criminal histories were common, often for burglary/theft and sexual offenses (Jeary, 2005; Lea et al., 2011; Safarik et al., 2002).

#### Victim-Perpetrator Relationship

Twenty-six studies disclosed victim-perpetrator relationships, with strangers being identified most often (*n* = 14), and reported as the majority most frequently (*n* *=* 8). Acquaintance relationships were also prevalent (*n* = 11), being the majority in five studies. Among institutional studies, dominant relationships included other residents (*n* = 2) and facility staff (*n* = 2), with Smith et al. (2019), noting equal rates (25% each). Though not a majority, other relationships included husband/spouse (*n* = 10), other family members (*n* = 9), and friends (*n* = 2).

#### Witnesses

Witnesses were infrequently reported (*n* = 8 studies), with involvement specified in less than 35% of incidents. The highest recorded percentage of cases involving at least one witness was noted by Teaster and Roberto (2004, 51.2%), most commonly facility staff and other residents within institutional settings.

### What Typically Occurs During Events of SVA Against Older People?

To assess what typically occurs during events of SVA against older people, data was extracted from applicable studies (*n* = 28) according to acts, violence, and obtained injuries.

#### Acts

Fourteen studies identified rape or penetration (digital, vaginal, or anal) as the most prevalent SVA act perpetrated against older victims. Additionally, fondling was repeatedly identified (*n* = 9), often more common among male victims (Budd & Liddic, 2021; Teaster & Roberto, 2004; Teaster et al., 2007). Other identified sexual acts included; sexualized kissing, indecent touching, exhibitionism, deliberate humiliation, verbal sexual abuse (e.g., sexual innuendo, jokes, or calls), molestation, and unwanted sexual interest in the victim’s body.

Seven studies indicated that additional crime took place alongside sexual assault, most often theft/property crime, particularly within community settings. Additionally, Budd and Liddic (2021) noted a small number of cases involved perpetrators consuming alcohol or drugs during perpetration, which was more prevalent in cases involving female victims (8%, compared to 6% of cases with a male victim). Upon completion of assault, Chopin and Beauregard (2020a) stated that 64.2% of victims were intentionally released, 16.1% escaped, and 11.5% were saved by a third party. Where the victim was killed, 25% of cases involved post-mortem activity (Chopin & Beauregard, 2020b), and the body was left uncovered 57% of the time (Safarik et al., 2002).

#### Violence and Injury

Ten studies provided information on non-essential use of violence, wherein violence was described as an act of victimization, rather than a strategy to enable or advance the offense. According to Ball and Fowler (2008), such violence was exercised in 31% of cases—a rate mostly consistent with other included samples (e.g., 21%, Almond et al., 2022; 30.8%, Lee et al., 2019). Beating and strangulation were evidenced in number of cases, as well as stabbing (Chopin & Beauregard, 2020a, 2020b), with several studies describing physical violence as excessive or extreme (Jeary, 2005; Lazar, 2019; Murphy & Winder, 2016).

Expectedly, numerous studies (*n* = 17) detailed injuries obtained during victimization. Many victims had obvious body trauma, ranging from 29.4% of the sample (Burgess & Phillips, 2006) to 65.6% (Del Bove et al., 2005). Common injuries included bruising, cuts/lacerations, tenderness, skin tears, swelling, fractures, and bleeding, often to the genital area or head/face/neck region (Almond et al., 2022; Baker et al., 2009; Del Bove et al., 2005). Victim death was recorded in five studies (Burgess & Phillips, 2006; Burgess et al., 2008; Jeary, 2004, 2005; Lazar, 2019), excluding those only considering sexual homicides (Chopin & Beauregard, 2020b; Safarik et al., 2002) In particular, Lazar (2019) found 27.5% of sampled cases occasioned death, though the nature of the data (cases finalized within the courtroom) may naturally include more serious incidents.

### Where Do Incidents of SVA Against Older People Most Commonly Occur?

Twenty-eight studies provided information on where SVA events typically occur. The victim’s home was most common (*n* *=* 17), with 12 studies reporting rates above 70% in these settings. Institutional locations were also prevalent, being identified in 20 studies, and marked as the majority in 10 (6 studies focused exclusively on institutional settings). Though less frequent, other locations included perpetrator residence, transport-related locations (e.g., bus stops and train stations), business locations, or public areas.

### When Do Incidents of SVA Against Older People Typically Occur?

Nine studies recorded time of offense. Of these, majority (*n* = 5) stated perpetration most often occurred during hours of darkness, primarily late evening or early morning, or while the victim slept (Chopin & Beauregard, 2020b). Nonetheless, SVA was also found to be perpetrated during daylight hours (*n* = 3), with Smith et al. (2019) finding majority of cases in institutional settings occurred between 4:00 am and 10:30 am.

### How Are SVA Offenses Perpetrated Against Older People?

Methods or strategies used to perpetrate SVA against an older person were detailed in 14 studies. These included type of approach and attack, control, use of weapons, and forensic awareness or planning.

#### Approach and Attack

Seven studies described the methods used by an offender to initially gain access to and overpower older victims. When gaining access to the assault location, two studies specified entry was often forced (Lea et al., 2011; Safarik et al., 2002), though entrance through unlocked doors and windows was also noted. Within institutional settings, Ramsey-Klawsnik et al. (2008) purport that many perpetrators had easy access to vulnerable victims through status as employees, residents, family, or visitors, illustrating the variability of methods across settings.

Two approach strategies are recognized within reviewed studies; “con” approaches are those where the offender misleads the victim to gain trust prior to attack, while “surprise” approaches involve the perpetrator waiting until the victim is in a vulnerable state before initiating an attack (Lea et al., 2011). Six studies detailed characteristics relevant to these methods. “Con” approaches (*n* = 5) were implemented roughly 30% to 45% of the time, with approximately 20% of cases involving victims inviting perpetrators into their residence on the basis of a ruse (Chopin & Beauregard, 2020b; Safarik et al., 2002). Five studies also specified strategies consistent with the “surprise” approach, with incidents often initiated while the victim slept (Almond et al., 2022; Chopin & Beauregard, 2020a, 2020b; Eckert & Sugar, 2008). Prevalence of “surprise” approaches varied, ranging from 13% (Eckert & Sugar, 2008) to 73% (Lea et al., 2011).

Where method of attack was reported (*n* *=* 3), blitz attacks (immediate use of force to incapacitate victim) were most prominent, particularly in cases of sexual homicide (Chopin & Beauregard, 2020b; Safarik et al., 2002).

#### Control

Twelve studies described techniques of coercion or control used throughout SVA events. Physical force or violence was identified most often (*n* = 8), with Nóbrega Pinto et al. (2014) reporting that 64% of cases utilized this method of control. Notably, Burgess and Phillips (2006) found that when a victim experienced dementia, physical beating was frequently used for control, whereas verbal threats were more prevalent in non-dementia cases. Nonetheless, threats were recorded across seven studies, regardless of victim cognition. Victim restraint was also reported (*n* = 6), generally demonstrated in 30% to 45% of cases (Chopin & Beauregard, 2020b; Del Bove et al., 2005; Eckert & Sugar, 2008), while mere presence of the offender and abuse of authority (*n* = 4) were prevalent control tactics in institutional settings.

#### Use of Weapons

Nine studies reported weapons use during victimization, with rates often lower than 8%. The key outlier was Chopin and Beauregard (2020b), who reported almost 70% of cases involved a weapon (however, this study explicitly considered cases of sexual homicide, suggesting potentially greater weapons involvement in offenses of this motive and nature). According to Budd and Liddic (2021) and Safarik et al. (2002), personal weapons (e.g., hands and feet) were favored, though studies also stated knives, sharp objects, rope, tape, handcuffs, hammers, and victims’ walking sticks were used.

#### Forensic Awareness and Planning

Six studies specified forensic awareness strategies and offense planning behaviors. Chopin and Beauregard (2020a) reported more than half (53%) of all perpetrators demonstrated forensic awareness, while Chopin and Beauregard (2020b) found over 20% destroyed evidence post-assault, likely to avoid detection. Lea et al. (2011) similarly noted that when offending against older people, perpetrators used significantly more sound precautions (e.g., placing a hand over the victim’s mouth to prevent calls for help). Nonetheless, two studies found SVA events were often opportunistic (Murphy & Winder, 2016; Safarik et al., 2002), with lack of planning conceivably evidenced in an absence of condom use, and traces of semen found in a high majority of cases (Chopin & Beauregard, 2020a; Nóbrega Pinto et al., 2014).

### Prevention Recommendations

Most studies (*n* = 23) contained prevention recommendations for SVA events involving older victims (see Supplemental Appendix B). Of these, 18 studies offered *primary* prevention strategies, including increased community awareness (Baker et al., 2009; Filipska et al., 2019; Fisher & Regan, 2006; Jeary, 2004; Lazar, 2019; Ramsey-Klawsnik et al., 2008; Smith et al., 2019), clearer policies (Alon et al., 2018; Filipska et al., 2019; Jordanova Peshevska et al., 2014), increased guardianship (Budd & Liddic, 2021; Burgess & Phillips, 2006; Payne & Gainey, 2006; Ramsey-Klawsnik et al., 2008), and bolstering of home security and community relationships, therefore enhancing safety (Budd & Liddic, 2021; Chopin & Beauregard, 2020a).

*Secondary* prevention strategies were identified in 15 studies, with recommendations predominately concerning the need for education and training on how to identify abuse and engage in early intervention (Alon et al., 2018; Burgess et al., 2008; Fisher & Regan, 2006; Lea et al., 2011; Lee et al., 2019; Nobels et al., 2021; Nóbrega Pinto et al., 2014; Payne, 2010; Qu et al., 2023; Ramsey-Klawsnik et al., 2008; Teaster & Roberto, 2004). Implementation of mechanisms to identify at-risk individuals, such as employee screening and background checks (Baker et al., 2009; Payne, 2010; Payne & Gainey, 2006; Ramsey-Klawsnik et al., 2008) and purposeful positioning of at-risk residents in institutional settings (Teaster & Roberto, 2004) were similarly recognized as possible prevention measures within literature. Additionally, both Alon et al. (2018) and Payne and Gainey (2006) suggested the need for improved working conditions within institutional settings to reduce caregiver stress and burden. As these authors found links between these symptoms and perpetration of abuse, working to relieve these attitudes may assist in preventing escalation of abuse for those at a higher risk of perpetrating.

## Discussion

This scoping review examined literature on SVA events perpetrated against older people to determine who is involved, what typically occurs, when and where incidents take place, and how offenses are perpetrated. Using Arksey and O’Malley’s (2005) scoping review framework, data was extracted and analyzed from 33 studies meeting inclusion criteria to provide a comprehensive overview of what is currently known about events of SVA involving older victims, how this knowledge informs prevention, and to identify key gaps in literature on this topic. Critical findings are summarized in Table 3.

**Table 3.** Summary of Critical Findings.

• After screening 1,278 records, 33 studies contained information relevant to the SVA criminal event involving adult victims • Reviewed sources primarily reported who is involved in incidents, what acts commonly take place, and where they usually occur. It was determined that older victims are most often female, while perpetrators are predominately male, generally younger than the abused. Rape or penetration was the most common form of abuse, with the victim’s home being the primary abuse location. • Significant gaps in knowledge were recognized: ○ Limited information was available around *when* SVA events typically occur and *how* they are perpetrated ○ Prevention recommendations were frequently identified within reviewed studies, however very few related to environmental factors influencing opportunity for offending. • Findings signify a need to more comprehensively understand the crime commission process of SVA perpetrated against older people to determine the situational circumstances of abuse, enabling targeted, and holistic, prevention of these crimes.

*Note*. SVA = sexual violence and abuse.

### Nature and Dynamics of SVA Perpetrated Against Older People

A review of literature has identified various characteristics that are common to SVA events involving older victims. Overall, events typically involved a White female victim and White male perpetrator, took place within the victim’s residence during hours of darkness, and comprised severe forms of violence and victimization. Rape or penetration was the most frequently identified type of assault, with physical violence being a primary method of control, occasioning significant harm and injury to the older person. Concerningly, several victims died during victimization, further demonstrating the extreme and violent nature of these offenses and the vulnerability of victims.

Literature establishes that, theoretically, older people present a low risk for victimization due to participation in more conservative routine activities, yet are often susceptible to violent forms of abuse (Bows, 2019; Georgoulis et al., 2024). Importantly, this review has identified a range of characteristics that may contribute to perceptions of vulnerability and risk, as per environmental criminology principles. Principally, victim limitations (e.g., cognitive or physical impairment) were identified in more than half of all included studies, with higher prevalence reported in institutional settings. As these ailments are more likely to develop in later life, potential offenders may perceive an older person to be more easily overpowered (due to frailty or disability) and less cognizant of abusive behavior occurring (due to cognitive impairment; Georgoulis et al., 2024; Ramsey-Klawsnik, 2003; Vierthaler, 2008). This premise aligns with Ramsey-Klawsnik’s (2003) proposition that impairment in older adulthood can significantly reduce capacity for self-protection, therefore potentially increasing vulnerability. Indeed, this review indicated an offender preference for personal weapons (e.g., hands and feet), supporting presumptions of older people being “easy” to overpower and exploit (Beauregard & Chopin, 2022). Nonetheless, it is recognized that not all victims were physically disabled or cognitively impaired, particularly within community settings, suggesting alternative vulnerability factors within these environments.

According to routine activity principles, risk can be mitigated through presence of capable guardianship within victims’ routine environments (Cohen & Felson, 1979; Felson, 2017). However, of the studies reporting living arrangements, most specified community-dwelling victims often lived alone, therefore invalidating benefits of guardianship and further increasing potential risk (Blundell et al., 2022). When considering the costs and benefits of perpetrating a sexual offense, the privacy provided in this setting may appeal to an offender through reduced risk of detection and disruption by a guardian (Beauregard & Chopin, 2022). This may then explain why “rape” or “penetration” and the “victim’s residence” were the most common event characteristics in reviewed studies—the concealed nature of this location may enable perpetration of more violent and invasive assaults. Guardianship was similarly recognized as an issue in care institutions, where Payne (2010) and Teaster et al. (2015) found less than 35% of cases were witnessed by a third party. Collectively, reduced capacity for self-protection and an absence of capable guardianship may dynamically influence an offender’s decision-making process, where the effort required to perpetrate the crime is low and risk of detection is significantly reduced, thus increasing opportunity for SVA perpetration.

Nevertheless, ageist attitudes propose older people cannot be SVA victims as they are not considered sexually active or attractive (Goldblatt et al., 2022). Instead, “real rape” is seen to only involve younger female victims, who are attacked at night by a sexually-motivated stranger (Bows, 2019; Bows & Westmarland, 2017; Lazar, 2019). Crucially, this review has found that many characteristics of “real rape” are mirrored in literature on SVA against older people (e.g., penetrative assaults, stranger relationships, and perpetration during darkness). Though this may be influenced by higher reporting of this abuse type (Bows & Westmarland, 2017), it is nonetheless a significant finding. Moreover, perpetrators were found to be comparatively younger than victims in many instances, emphasizing age is no barrier to sexual victimization, including the most severe forms of abuse. Taken together, these findings work to dispel rape myths and combat ageist stereotypes, further recognizing SVA as a pervasive and malevolent issue affecting people young and old.

### Gaps in Knowledge

This scoping review has identified significant gaps in knowledge and understanding of SVA events involving older victims. Principally, definitions of “older” varied substantially with five separate minimum age classifications applied, ranging from 50 years of age to 70 years of age. Though there is no “typical” older person (WHO, 2022), definitional differences create difficulties when synthesizing and comparing literature (Dong, 2015; Moir et al., 2017), therefore warranting the need for consistent operationalization within research methodologies. It is also recognized that the lifestyle and abilities of a 50-year-old generally differ from those of a 70-year-old person. Broad categorizations of “old age” may not capture risk variation across older adulthood, therefore affecting the applicability and effectiveness of responses.

For example, the Organization for Economic Co-operation and Development (OECD, 2021) reports average retirement age within OECD countries is 63.8 years for males and 62.4 years for females. Prior to retirement, adults commonly engage in regular hours of employment, therefore influencing routine activities and lifestyle. Once retired, these routines likely undergo significant alteration, perhaps seeing more time spent at home, consequently changing risk exposure (Chopin & Beauregard, 2020a; Lea et al., 2011). Moreover, the ARC Centre of Excellence in Population Ageing Research (2018) reports that between the ages of 70 and 84, rates of dementia double every 5 years, further demonstrating the advancement of cognitive and physiological changes in later life. While this review identified a range of commonalities across studies regardless of minimum victim age, the way age is defined and measured may impact interpretation of results and applicability of research implications. Future research comparing vulnerability and risk throughout older adulthood, or comparing characteristics of SVA across the aging process, may add an additional lens to this emerging topic, enhancing understanding and strengthening response.

Additionally, use of theory to inform research in this field is noticeably absent. Only six studies incorporated a theoretical framework, accounting for just 18% of all included literature. Of these, four (12%) used environmental criminology perspectives, with recurring authorship noted within this subset (Chopin & Beauregard, 2020a, 2020b; Payne, 2010; Payne & Gainey, 2006). Though not all research requires a theoretical basis, it is argued this practice is particularly beneficial for criminology research as it can help inform research questions and guide practice, while also playing a significant role in interpretation of results (Bachman & Schutt, 2008). In this sense, theory may help to organize and understand outcomes, ultimately enhancing the validity and applicability of empirical findings, thus contributing to the development of evidence-based conclusions (Bachman & Schutt, 2008). Criminological theory is distinctly absent from this field of research, yet, as this review evidenced, environmental criminology perspectives have merit for understanding contextual vulnerability and risk factors, as well as offender decision-making processes. As SVA is a criminal issue, future research must draw on relevant theories, such as environmental criminology, to better understand SVA events perpetrated against older people, therefore enhancing interpretations and possible prevention efforts.

When assessing the criminal event itself, to date, considerable attention has been given to understanding victim and perpetrator characteristics, as well as involved acts and incident outcomes. Yet, much less is known about *when* these offenses occur and *how* they are perpetrated, including what precipitates incidents and what opportunity structures enable SVA. Only nine studies contained information on time of offense, with most providing broad categorizations (e.g., daylight or darkness). Recurring recognition of “darkness” as a time of victimization sees theoretical understandings of risk and vulnerability further applied to SVA incidents involving older victims. Situational explanations and responses to crime persistently note that decisions to offend are often influenced by perceived or actual surveillability (Armitage, 2017; Ekblom, 2011). The cover of darkness (along with an absence of guardianship) may therefore negatively impact natural surveillance opportunities, ultimately reducing perceived risk for an offender. Moreover, most people sleep at night. Indeed, several reviewed studies specified SVA being initiated while the victim slept (Chopin & Beauregard, 2020a, 2020b; Eckert & Sugar, 2008), signifying a time when the victim is in a considerably vulnerable state and likely unable to defend themselves against an attack. Nonetheless, little to no information is provided on specific times and days when these offenses are most likely to occur, which is a gap that future research should address. Having deeper insight into these characteristics may identify particular times where additional protections are needed to deter perpetrators, as per crime pattern theory principles, generating targeted prevention and response (Brantingham et al., 2017; Eck & Weisburd, 1995).

Moreover, there was notable variance in the methods and strategies used to successfully perpetrate SVA offenses. Though clear patterns were identified concerning frequent physical violence to control victims and minimal weapon usage, information surrounding offense preparation, initial interactions, progression of offending, and incident conclusion were less distinct. For example, only two studies discussed offense planning (Murphy & Winder, 2016; Safarik et al., 2002), with both suggesting incidents were opportunistic. Likewise, six studies reported approach characteristics, though results varied, with strategies consistent with “surprise” and “con” styles described in five studies each. Knowing more about how these offenses unfold, along with the opportunity structures enabling perpetration, may then help to direct prevention efforts toward higher-risk environments, therefore increasing safety and reducing vulnerability. Moreover, very little is known about progression and completion of offending, with few references to forensic awareness and evidence destruction (Chopin & Beauregard, 2020a, 2020b; Lea et al., 2011; Safarik et al., 2002), signaling an area of research that should be prioritized.

Most studies included prevention recommendations, with a slight majority advocating for primary initiatives. Yet, at both levels, strategies were predominately people-focused (e.g., increased awareness, education, and training on how to recognize and respond to potential abuse, and stricter screening and supervision of staff in institutional settings). Comparatively, very few studies considered the environment in which abuse occurs, with only a small portion making SCP recommendations to deter potential offending (e.g., Budd & Liddic, 2021; Burgess & Phillips, 2006; Chopin & Beauregard, 2020a; Payne & Gainey, 2006; Ramsey-Klawsnik et al., 2008). Of these, most related to increasing guardianship and bolstering home security, demonstrating narrow application of SCP principles. Consequently, future research should more stringently consider the situational determinants of SVA events involving older victims to develop a comprehensive prevention framework that addresses both individual and environmental factors at primary and secondary levels.

### Limitations

Though the methods used throughout this scoping review ensured a comprehensive analysis of extant literature, limitations must be addressed. It is recognized that results were refined to any study published in English after the year 2000. This was conducted to ensure a breadth of literature was captured, while still remaining current. Nonetheless, relevant papers published prior to this date or in other languages were not included, having the potential to influence findings within this review. Moreover, it is acknowledged that there is no universal definition of SVA perpetrated against older people, with policies and procedures differing across cultures, jurisdictions, and research methodologies (Bows, 2018; Hand et al., 2022). Literature also establishes that SVA among older people is significantly underreported, particularly within institutional settings where reporting mechanisms (e.g., identification of abuse and staff discretion) may constrain disclosures (Smith et al., 2019). Similarly, additional complexities around reporting intrafamilial abuse (Gill, 2022) and sociocultural norms influencing perceptions of sexual violence (Hand et al., 2022) may have contributed to a disproportionate number of analyzed cases involving White, female victims who had been raped by a stranger. Resultingly, characteristics identified within this review may not be reflective of the significant number of SVA cases that are not recognized and reported, instead representing a potentially limited subgroup of victims and abuse types (Bows & Westmarland, 2017; Fileborn, 2017).

Likewise, aims and measures differed across the study sample, therefore influencing data extraction; interpretations could only be based on information reported within the literature. It was not always possible to distinguish between community and institutional abuse incidents in studies using a “mixed” setting, however Georgoulis et al. (2024) suggest incidents in residential care facilities may involve different dynamics and vulnerabilities. Therefore, the contextual nuances of SVA perpetration in these settings may not have been adequately captured in this review. Individual study limitations are also recognized as impacting the reliability of findings. Nonetheless, this review followed rigorous protocols and had multiple reviewers conducting each stage of screening and extraction, therefore minimizing bias and ensuring interpretations were robust.

## Conclusion

According to Wikstrom (2007) and Clarke (2017), effective prevention strategies must be underpinned by a comprehensive understanding of the mechanisms and causes of specific crimes (i.e., how and why they are perpetrated). Yet, limited empirical attention has been paid to formulating a criminological understanding of SVA against older people. As this review has evidenced, environmental criminology is a unique and beneficial lens through which to explore this issue. This approach considers the interplay between victim vulnerability, offender decision-making, and environmental conditions in shaping and facilitating abuse incidents, focusing on how these elements come together in time and place. Integrating knowledge of the individual-level and environmental factors contributing to offense perpetration can then diversify opportunities for situation-focused prevention, highlighting the importance of proactively addressing risk and vulnerability within the context of SVA against older victims. Consequently, the results of this review demonstrate the suitability and merit of environmental criminology in enriching current understandings, enhancing preventative efforts, and directing future research in this field.

Table 4 summarizes advancements in practice, policy, and research in this regard.

**Table 4.** Summary of Implications for Practice, Policy, and Research.

• Cognitive and physical impairment, along with living alone, are commonly identified characteristics among older SVA victims, suggesting the need for increased social support and guardianship to protect against abuse. • Rape or penetration were the most prevalent abuse acts identified, possibly due to a higher likelihood of this type of abuse being reported. Education and awareness around various types of SVA should be prioritized among the older population to ensure all forms of SVA are recognized and appropriately reported, regardless of severity. • A universal definition for “older age” is needed to ensure consistency across study methodologies and enhance synthesis of findings. • As aging is a non-linear process, and most studies implement broad categorizations of “old age” (e.g., anyone over 60 years), future research should assess the variability of risk and vulnerability across older adulthood to determine whether there are differences throughout the aging process. • Future research on SVA perpetrated against older people should be informed by criminological theory, such as environmental criminology perspectives, and seek to gain a more comprehensive understanding of crime commission processes (i.e., how these crimes are perpetrated). This may then lead to identification of more diverse and proactive prevention strategies.

*Note*. SVA = sexual violence and abuse.

Overall, studies routinely report information relating to who is involved and what takes place during victimization, yet much less is known about when and how these offenses are perpetrated. Though a small percentage of studies described the methods and strategies used to successfully perpetrate these crimes, there were often no distinct patterns, and very limited insight into how offenses were initiated, proceeded, and completed. Moreover, criminological theory and situation-focused prevention recommendations were distinctly lacking, emphasizing the need for more empirical attention and theoretical application within this space to enhance understanding. A more comprehensive understanding of the processes involved in offense perpetration and the circumstances enabling and advancing incidents is therefore warranted to establish a more complete picture of this issue and identify diverse opportunities for prevention and intervention of SVA events against older people.

## Supplemental Material

sj-docx-1-tva-10.1177_15248380241265387 – Supplemental material for A Scoping Review of Sexual Violence Events Perpetrated Against Older People Supplemental material, sj-docx-1-tva-10.1177_15248380241265387 for A Scoping Review of Sexual Violence Events Perpetrated Against Older People by Madeline Lee, Nadine McKillop and Emily Moir in Trauma, Violence, & Abuse

## Notes

1. Calculations based on WHO (2022) population estimates.

2. Only the first five pages of results (50 records) were exported from Google Scholar, as relevancy declined significantly after this point.
