# Supplementary material for: A Scoping Review of Sexual Violence Events Perpetrated Against Older People
Source: Trauma Violence Abuse. 2024 Jul 31;25(5):3951–66. doi: 10.1177/15248380241265387 (PMC11545133; doi:10.1177/15248380241265387)
Supplement: sj-docx-1-tva-10.1177_15248380241265387 – Supplemental material for A Scoping Review of Sexual Violence Events Perpetrated Against Older People [file sj-docx-1-tva-10.1177_15248380241265387.docx]

**Supplemental Appendices**

**Supplemental Appendix A:** Predominate SVA event characteristics

| **Citation** | **Who** | **What** | **Where** | **When** |  | **How** |
| --- | --- | --- | --- | --- | --- | --- |
| Almond et al. (2022) | **Victims**: All female, 60-101yrs (*M=*73yrs), 92% White.  **Perpetrators** (*n*=68): All male, 14-63yrs (*M=*32yrs), 71% White. 74% had prior convictions.  **Relationship**: 85% stranger. | **Acts:** 36% disrobement. 29% had property stolen.  **Violence/Injury**: 21% involved violence. Force often minimal (24%), with facial injuries common (22%). | 79% indoors, 22% outdoors | 64% darkness, 35% daylight |  | **Approach/Attack:** 55% surprise, 45% confidence |
| Alon et al. (2018) | **Victims:** 98% female  **Relationship:** 75% spouse | **Acts:** 63% sexual assault, 19% indecent assault | 36% nursing homes |  |  |  |
| Baker et al. (2009) | **Victims:** All female, 50-98yrs (*M=*62yrs), 70.2% White. 53% had dementia, 21% physically disabled.  **Perpetrators:** 77% male, 2% female.  **Relationship:** 75% known to victim. | **Violence/Injury:** 38% had body trauma, 20% genital trauma. | 35% domestic, 29% institutions. |  |  | **Control:** Physical violence, victim restraint, and threats used.  **Weapons:** 17% of non-institutional cases involved a weapon. |
| Ball and Fowler (2008) | **Victims:** All female.  **Perpetrators:** All male, mid-teens to mid-70s (*M=*57yrs). 94% offended alone. | **Acts:** 43% rape, 12.5% groped.  **Violence/Injury:** 31% involved violence, 6% had bruising. | 81% domestic location. | 56% between 6am-6pm |  | **Control:** Force/coercion was used in 12.5% of cases. |
| Bows and Westmarland (2017) | **Victims:** 92% female, 60-98yrs, mostly White.  **Perpetrators:** 85% male, 66% under 60yrs, mostly White.  **Relationship:** 26% acquaintance, 20% stranger. | **Acts:** 72% rape, 28% assault by penetration | 54% victim's home, 21% care home. |  |  |  |
| Budd and Liddic (2021) | **Victims:** 85% female, 60-99yrs, majority White.  **Perpetrators:** Mostly male, 10-99yrs, majority White. Often offended alone.  **Relationship:** Mostly extrafamilial. | **Acts:** 40% of females were raped, 27% of males were fondled. Additional violent and property crime occurred in some cases.  **Violence/Injury:** Injury often minor. | Mostly private settings. |  |  | **Control:** Force common (approx. 70%), mostly physical.  **Other:** Perpetrator substance use less common with older victims. |
| Burgess and Phillips (2006)^1^ | **Victims:** 91.9% female, 56-100yrs, 80% White. Limitations were common (60.7% physical, 60% cognitive).  **Relationship:** 27% strangers, 26% incestuous.  **Witnesses:** 32% of cases had an eyewitness. | **Violence/Injury:** 29.4% had external injuries. 4 victims died. | 72.6% own / family home, 22.6% institutions |  |  | **Control:** 43.3% by offender’s presence. 62.5% with dementia were beaten, 57.6% non-dementia victims verbally threatened.  **Weapons:** 3.9% involved a weapon. |
| Burgess et al. (2008)^1^ | **Victims:** 93.5% female, 60-100yrs (*M=*78yrs), 82.3% White.  **Perpetrators:** 90.9% male, 13-90yrs. Limitations common, higher in APS group (65.7% physical, 70.5% cognitive).  **Relationship:** 26.1% strangers, 23.3% other family.  **Witnesses:** 15% of cases had a witness. | **Acts:** Kissing, fondling, penetration.  **Violence/Injury:** 59% genital, 18% anal injuries. 4 victims died. | 72% private homes, 23.2% facilities. |  |  | **Control:** 43.3% by offender’s presence.  **Weapons:** 3.9% involved a weapon. |
| Cannell et al. (2014) | **Victims:** 50.2% female, 60-99yrs, 72.9% White. | **Acts:** 53.4% non-contact, 43.1% contact. |  |  |  |  |
| Chopin and Beauregard (2020a) | **Victims:** 98.5% female, 65-94yrs (*M=*77yrs). 20.7% physically or psychologically disabled.  **Perpetrators:** *M=*33.3yrs. 40.8% presented paraphilias, 13.6% often engaged in crime.  **Relationship:** 74.6% stranger  **Witnesses:** 26.9% were witnessed. | **Acts:** 56.9% vaginal intercourse, 43.8% fondling. 64.6% of victims were intentionally released.  **Violence/Injury:** 30% beaten. 19.2% had severe injuries. | 71.5% at a residence | During domestic activities |  | **Approach/Attack:** 40.7% con, 31.5% surprise. 38.4% blitz attack.  **Weapons:** 18.4% involved presence of a weapon.  **Forensic Awareness:** Demonstrated in 53% of incidents. |
| Chopin and Beauregard (2020b) | **Victims:** 89.3% female  **Relationship:** 50% acquaintance | **Acts:** 48.2% vaginal intercourse, 23.2% foreign object insertion. Items taken in 50% of cases.  **Violence/Injury:** 50% beaten, 42.8% strangled | 73.2% victim residence | 23.2% assaulted while sleeping |  | **Approach/Attack:** 44.6% blitz, 33.9% con.  **Control:** 30.3% involved restraints.  **Weapons:** 69.6% involved weapons.  **Forensic Awareness:** 21.4% destroyed evidence. |
| Del Bove et al. (2005) | **Victims:** All female, 55-87yrs (*M=*65yrs). 86% White. 42.6% lived alone. 19.7% cognitively disabled.  **Relationship:** 47.5% acquaintance, 42.4% stranger. | **Acts:** 65% vaginal penetration, 15% fondling.  **Violence/Injury:** 65.6% had physical trauma. | 51.7% victim's home, 5% institutions. |  |  | **Control:** 45.9% physical restraint, 26.2% threats, 19.7% physical violence.  **Weapons:** 6.6% of cases involved weapons. |
| Eckert and Sugar (2008) | **Victims:** All female, 55-98yrs. 33% were physically disabled.  **Perpetrators:** 64.7% offended alone.  **Relationship:** 23.5% acquaintance, 18.6% stranger.  **Witnesses:** 6.9% were referred by a witness. | **Violence/Injury:** 38.3% had body trauma, 35.6% genital trauma. | 36.3% victim's home, 33.3% care facility. |  |  | **Approach/Attack:** 54.9% of victims were impaired at onset. 13.7% asleep.  **Control:** 34.3% restrained, 11.8% threatened.  **Weapons:** 7.8% of cases involved a weapon. |
| Filipska et al. (2019) | **Victims:** 82.4% female. 52.9% aged 60-65yrs. |  |  |  |  |  |
| Fisher and Regan (2006) | **Victims:** All female.  **Relationship:** 73% spouse/boyfriend. | **Acts:** 85% of SVA victims were pressured to have sex. |  |  |  |  |
| Jeary (2004) | **Victims:** Mostly female (RC) settings. Mostly aged 70s-80s (IL).  **Perpetrators:** All male (RC); either gender (DC); 18-35yrs (IL).  **Relationship:** Residents and staff (RC), spouses, sons, (DC), mostly strangers (IL). | **Acts:** Indecent touching through to attempted rape (RC). Majority involved burglary (IL).  **Violence/Injury:** Most physically injured, with excessive force and violence used. 8 victims died. (IL) | One-third of cases in RC settings. Mostly 'elderly people's' accommodation in IL settings. | Examples of assaults at night or early morning |  |  |
| Jeary (2005) | **Victims:** 60-98yrs, predominately female.  **Perpetrators:** All male, 16-70+yrs. Prior SVA convictions common for SA/Rape sample.  **Relationship:** mostly strangers (SA/Rape), mostly relatives, acquaintances, care workers (IA, SH). | **Abuse types:** Rape/attempted rape (*n*=20), indecent assault (*n*=20)  **Violence/Injury:** Excessive violence, resulting in severe injuries. | Commonly victim’s home, but also residential care settings. | SA/Rape: Late evening or early morning. |  | **Control:** Victim's clothing used to restrain. Physical force frequent.  **Weapons:** Weapons often used. |
| Jordanova Peshevska et al. (2014) | **Victims:** All female | **Acts:** Of the 1.3% reporting SA, rape/attempted rape most frequent (0.3% each). |  |  |  |  |
| Lazar (2019) | **Victims:** All female, 60-96yrs. 13% cognitively impaired. 70% lived in their own homes, mostly alone.  **Relationship:** 68% strangers, 12% acquaintances. | **Acts:** Rape, aggravated sexual assault. Majority occurred in the context of a home invasion.  **Violence/Injury:** Most involved extreme physical violence causing serious injuries and death (*n=*30). | Most rapes occurred in victims’ homes. |  |  |  |
| Lea et al. (2011) | **Victims:** 60 -92yrs (*M=*77yrs), 97% White  **Perpetrators:** 94% White. Previous convictions for property, theft and sexual offences.  **Relationship:** Majority were strangers. | **Acts:** Rape (*n*=19), attempted rapes or lesser SA (*n=*34). 40% involved theft. | 98% of indoor locations were the victim’s home | Majority occurred at night. |  | **Approach/Attack:** 73% surprise, 27% con.  48% involved forced entry.  **Forensic Awareness:** Significantly more sound precautions when victim was older. |
| Lee et al. (2019) | **Victims:** 94.9% female, 70-96yrs (*M=*83yrs). All White. 58% frail, 61.5% had dementia.  **Perpetrators:** All male, 22-87yrs (*M=*51yrs). 35.9% White.  **Relationship:** 28.2% strangers, 25.6% unrelated care-giver. | **Acts:** 43.6% penile-vaginal assault.  **Violence/Injury:** 30.8% involved physical violence. 28.2% had genital injury. | 38.5% client’s home, 25.6% residential care home. |  |  | **Control:** Threatening language used in 15.4% of cases. |
| Murphy and Winder (2016) | **Victims** (*n=*6): All lived alone.  **Perpetrators:** All male, 20-46yrs (*M=*30yrs), majority White.  **Relationship:** 40% acquaintances, 20% strangers. | **Acts:** All were convicted of rape.  **Violence/Injury:** All used high brutality and significant violence. | All occurred at the victim's home. |  |  | **Planning:** Opportunistic |
| Nobels et al. (2021) | **Victims:** 6% female (*n=*4), 9% male (*n=*3). Most depended on others for care. | **Acts:** Hands-off offences (*n=*3), hands-on offences (*n=*4). |  |  |  |  |
| Nóbrega Pinto et al. (2014) | **Victims:** All female, 66-91yrs (*M=*77yrs). 70% lived alone. 45% had a physical or mental handicap.  **Perpetrators** (*n*=13): All male, 17-81yrs (*M=*48yrs). Four had prior convictions.  **Relationship:** 57% known, 36% strangers.  **Witnesses:** 1 case only. | **Acts:** 50% vaginal penetration by penis.  **Violence/Injury:** Bruises, abrasions, and lacerations. | 58% victim's home. |  |  | **Control:** Physical violence used in 64% of cases. |
| Payne and Gainey (2006) | **Victims:** 36% cognitively impaired. | **Acts:** 54% hands-on offences; 25% harmful genital contact. | Nursing home sample only |  |  |  |
| Payne (2010) | **Victims:** Two-thirds female, 44.9% cognitively impaired.  **Perpetrators:** 78.4% male. One-third were repeat offenders.  **Relationship:** 68.9% aides, 18.4% 'other'.  **Witnesses:** 13.4% of cases had a witness. | **Acts:** Vast majority involved harmful genital contact. | Only health-care settings included |  |  |  |
| Qu et al. (2023) | **Victims:** Females more than twice as likely to experience SVA.  **Perpetrators:** 79% male. 35.2% had problems with alcohol, 29.7% had mental health problems.  **Relationship:** 41.5% friend. |  |  |  |  |  |
| Ramsey-Klawsnik et al. (2008) | **Victims:**77% female, 60-101yrs (*M=*79yrs), 86% White. 64% had dementia, 38% physically disabled.  **Perpetrators** (*n*=119): 78.4% male, 19-96yrs (*M=*56yrs). 60% White, 23% Black. Six had criminal histories.  **Relationship:** 43% staff, 41% other residents. | **Acts:** Molestation (*n=*57), vaginal rape (*n=*14, and *n*=7 attempted). | 73% nursing homes. |  |  | **Approach/Attack:** Easy access to vulnerable individuals via employment or residence.  **Control:** Six cases involved threats. |
| Safarik et al. (2002) | **Victims:** All female, *M=*77yrs. 86% White.  **Perpetrators:** 15-58 years. 44% White, 42% Black. 90% had criminal records.  **Relationship:** 52% known, 42% unknown. | **Acts:** 92% rape. Property removed in 72% of cases.  **Violence/Injury:** Cause of death most commonly strangulation (63%). Injuries often severe, some excessive. | 94% victim residence | 66% between 8pm and 8am |  | **Approach/Attack:** Forced entry in 37% of cases. 82% used a blitz attack.  **Weapons:** Personal weapons most common.  **Planning:** Little or no planning; spontaneous. |
| Smith et al. (2019) | **Victims:** All female, 65-100yrs (*Mdn=*83yrs). 73.9% had dementia.  **Perpetrators** (*n*=15): All male.  **Relationship:** 25% direct care staff, 25% other residents. | **Acts:** 60.7% vaginal contact/penetration.  **Injuries**: Bruising, skin tears, redness/swelling. | 28.6% in victim's bedroom. | Majority (17.9%) occurred between 4:00am-10:30am. |  |  |
| Teaster and Roberto (2004) | **Victims:** 95% female. 50% needed assistance with ambulation.  **Perpetrators** (*n*=76): 98% male, 19-89yrs. 28% had untreated psychiatric illness, 6.3% had criminal histories.  **Relationship:** 68.8% residents (in facility cases).  **Witnesses:** 51.2% of cases had a witness. | **Acts:** 73.2% sexualized kissing and fondling. | 76.8% in facilities, 15.9% at victim's home. |  |  |  |
| Teaster et al. (2007) | **Victims:** All male, 50-93yrs (*M=*71yrs). 73% White. 35% had communication difficulties.  **Perpetrators:** (*n*=24), 54% male, 42% female, 18-79yrs(*M=*36yrs), 46% White.  **Relationship:** 75% staff, 25% other residents.  **Witnesses:** 38% had no witnesses. | **Acts:** 35% fondling. | Nursing home sample only |  |  |  |
| Teaster et al. (2015) | **Victims:** All female, 66-101yrs (*M=*81yrs). 89% White. 70% had dementia, 27% physically disabled.  **Perpetrators** (*n*=60): 83% male, 19-95yrs (*M=*57yrs). 65% White. 20% cognitively disabled, 3 had criminal histories.  **Relationship:** 42% residents, 40% staff.  **Witnesses:** 64% did not have a witness. | **Acts:** 81% hands-on offences. | Nursing home sample only |  |  |  |
| ^1^Studies used similar samples, though analyzed different dimensions of SVA, hence both are included.  RC = residential care, DC = domiciliary care, IL = independent living, SA = sexual assault, IA = indecent assault, SH = sexual harassment | | | | | | |

**Supplemental Appendix B:** Prevention recommendations

| **Citation** | **Primary** | **Secondary** |
| --- | --- | --- |
| Alon et al. (2018) | - Clear policies and reporting systems - Supportive work environments to encourage discourse when dealing with SVA and discussions around handling incidents | - Screening tools for proper detection of abuse - Staff awareness and training on how to detect and respond to abuse |
| Baker et al. (2009) | - Education about SVA to guard against inadvertently creating environments where abuse can be perpetrated | - Employment history and criminal background checks - Immediate response to protect victim and preserve evidence |
| Budd and Liddic (2021) | - Bolster home security efforts - Increase social cohesion in neighborhoods, where neighbors can act as guardians |  |
| Burgess and Phillips (2006) | - Increased home visits and observation by care providers | - Immediate reporting of abuse and physical examination |
| Burgess et al. (2008) |  | - Raising caregiver awareness of SVA to recognize signs and implement response/intervention |
| Cannell et al. (2014) | - Health promotion efforts targeted toward older adults, encouraging them to seek services after exposure to sexual abuse |  |
| Chopin and Beauregard (2020a) | - Small changes in victim’s habits - Strengthening informal control through neighborhood cohesion, increasing potential for witnesses |  |
| Filipska et al. (2019) | - Increased awareness around the scope of the issue - Implementation of policies relevant to multiple abuse types |  |
| Fisher and Regan (2006) | - Awareness of health implications and the importance of identification and training for health care and service providers | - Training health care providers to look for signs of abuse |
| Jeary (2004) | - Increased awareness of SVA to prompt further avenues of exploration - Need for holistic assessment and routine reviews of care patients | - Need for effective selection, supervision and training of employees |
| Jordanova Peshevska et al. (2014) | - Integration of prevention into social and educational policy - Educational campaigns to promote gender and social equality of older people |  |
| Lazar (2019) | - Community projects to raise awareness of SVA among older people and promote access to legal and social services |  |
| Lea et al. (2011) |  | - Awareness of SVA to increase identification of older victims and enhance services |
| Lee et al. (2019) | - Greater inter-agency collaboration and awareness - Collaboration with minority populations to enhance recognition and reporting, tailoring services to be culturally sensitive | - Clearer referral pathways to reduce delays in recognition - Training to spot signs of intentional injury |
| Nobels et al. (2021) |  | - Training and detection tools to better recognize and respond |
| Nóbrega Pinto et al. (2014) |  | - Increased awareness of possible SVA indicators and early evaluation to detect abuse |
| Payne and Gainey (2006) | - Hiring and training of place managers to control environments and monitor potential offenders and victims - Increased guardianship and working in teams to ensure witnesses | - Empowering place managers to report problems - Better applicant screening (e.g., criminal background checks, psychological evaluations) |
| Payne (2010) | - Policies specifying the need for guardianship | - Strict hiring procedures - Specific training to recognize signs of abuse and understand reporting requirements |
| Qu et al. (2023) |  | - Raising awareness to improve identification and response - Systematic screening for abuse - Implementing mechanisms to identify those at risk of experiencing or perpetrating abuse |
| Ramsey-Klawsnik et al. (2008) | - Supervision of both employees and residents. - Awareness of the potential for visitors and family to assault patients | - Screening of potential employees and residents for evidence of dangerousness (e.g., background and reference checks, interviewing) - Staff training to recognize and respond to indicators of abuse - Swift reporting and investigation of suspected abuse |
| Smith et al. (2019) | - Staff awareness of SVA and duty to report alleged incidents |  |
| Teaster and Roberto (2004) | - Educate residents on what constitutes sexual abuse | - Staff training in detecting SVA and assisting victims (e.g., reporting pathways, intervention strategies) - Position at-risk residents near nurses’ stations. |
| Teaster et al. (2015) | - Collaborative provision of safe, high-quality care for residents |  |
